# The structure of *Serratia marcescens* Lip, a membrane-bound component of the type VI secretion system

**DOI:** 10.1107/S0907444911046300

**Published:** 2011-11-18

**Authors:** Vincenzo A. Rao, Sharon M. Shepherd, Grant English, Sarah J. Coulthurst, William N. Hunter

**Affiliations:** a College of Life Sciences, University of Dundee, Dundee DD1 5EH, Scotland

**Keywords:** β-sandwich, Gram-negative pathogens, lipoproteins, protein secretion, transthyretin, virulence

## Abstract

Lip is a membrane-bound lipoprotein and a core component of the type VI secretion system found in Gram-negative bacteria. The structure of a Lip construct (residues 29–176) from *Serratia marcescens* (*Sm*Lip) has been determined at 1.92 Å resolution. Experimental phases were derived using a single-wavelength anomalous dispersion approach on a sample cocrystallized with iodide. The membrane localization of the native protein was confirmed. The structure is that of the globular domain lacking only the lipoprotein signal peptide and the lipidated N-terminus of the mature protein. The protein fold is dominated by an eight-stranded β-sandwich and identifies *Sm*Lip as a new member of the transthyretin family of proteins. Transthyretin and the only other member of the family fold, 5-hydroxyisourate hydrolase, form homo­tetramers important for their function. The asymmetric unit of *Sm*Lip is a tetramer with 222 symmetry, but the assembly is distinct from that previously noted for the transthyretin protein family. However, structural comparisons and bacterial two-hybrid data suggest that the *Sm*Lip tetramer is not relevant to its role as a core component of the type VI secretion system, but rather reflects a propensity for *Sm*Lip to participate in protein–protein interactions. A relatively low level of sequence conservation amongst Lip homologues is noted and is restricted to parts of the structure that might be involved in interactions with physiological partners.

## Abstract 2

The high-resolution crystal structure of *S. marcescens* Lip reveals a new member of the transthyretin family of proteins. Lip, a core component of the type VI secretion apparatus, is localized to the outer membrane and is positioned to interact with other proteins forming this complex system.

## 1. Introduction

Protein secretion systems are critical to the virulence and host-interaction processes of Gram-negative pathogens. Different bacterial species possess different combinations of one or more specialized proteinaceous machines that secrete toxins, adhesins, hydrolytic enzymes and proteins able to manipulate eukaryotic signalling pathways (Gerlach & Hensel, 2007 ▶; Holland, 2010 ▶). The most recently discovered system, the type VI secretion system (T6SS), is present in many Gram-negative bacteria and is implicated in virulence in important human pathogens including *Pseudomonas aeruginosa* (Cascales, 2008 ▶; Filloux *et al.*, 2008 ▶; Jani & Cotter, 2010 ▶). It has also been shown to contribute to the virulence of economically significant animal and plant pathogens (Liu *et al.*, 2008 ▶; Blondel *et al.*, 2010 ▶; Sarris *et al.*, 2010 ▶). Some T6SSs appear to target other bacterial cells instead of, or in addition to, eukaryotic cells (Hood *et al.*, 2010 ▶; MacIntyre *et al.*, 2010 ▶; Murdoch *et al.*, 2011 ▶). This suggests that T6SSs may contribute to allowing pathogens to proliferate in polymicrobial infection sites and/or to persist in different environmental reservoirs (Schwarz *et al.*, 2010 ▶; Murdoch *et al.*, 2011 ▶). *Serratia marcescens* is an opportunistic pathogen, a significant cause of hospital-acquired infections and an important reservoir of antibiotic-resistance determinants in the clinical environment (Hejazi & Falkiner, 1997 ▶). It is also a tractable model organism in which to dissect the structure–function relationships in the T6SS (Murdoch *et al.*, 2011 ▶).

Studies of the T6SS have started to reveal information on the components and the biological role of this recently discovered system (Cascales, 2008 ▶; Filloux *et al.*, 2008 ▶; Pukatzki *et al.*, 2009 ▶; Bönemann *et al.*, 2010 ▶). T6SSs are large multiprotein complexes encoded on variable gene clusters characterized by the presence of genes encoding 13 ‘core’ components. These are thought to form the basic secretion apparatus, which is coupled with ‘accessory’ components that are conserved across many or only a few systems. Key core components include the putative extracellular Hcp/VgrG assembly, which is thought to form a cell-puncturing device similar to that of bacteriophage tail structures (Pukatzki *et al.*, 2009 ▶). There are a number of predicted cytoplasmic proteins (*e.g.* an ATPase called ClpV) and several inner membrane proteins (*e.g.* IcmF and IcmH). Additionally, and the subject of this work, the only outer membrane component reported to date is a periplasmic-facing outer membrane lipoprotein (Lip; Aschtgen *et al.*, 2008 ▶).

Genetic studies indicate that in *S. marcescens* this lipoprotein (*Sm*Lip) makes an essential contribution to the basic function of the T6SS and to T6SS-dependent antibacterial killing activity (Murdoch *et al.*, 2011 ▶). We now report the high-resolution structure of *Sm*Lip determined following phase determination using single-wavelength anomalous dispersion (SAD) measurements based on the scattering properties of iodide ions. The localization of the protein in *S. marcescens* itself and bacterial two-hybrid data are reported to investigate the propensity for self-association. The structure reveals a remarkable similarity to transthyretin, a vertebrate hormone-distribution protein, and comparisons suggest which parts of *Sm*Lip may be involved in protein–protein interactions with partner components of the T6SS.

## 2. Methods

### 2.1. Protein expression and purification

The *S. marcescens lip* gene (*SMA2252*; Murdoch *et al.*, 2011 ▶) encoding amino-acid residues 30–176 was amplified from genomic DNA (strain Db10) using the forward primer 5′-­catatgGCCAAAAGCGTGCCGTCGCGTTACAG-3′ and the reverse primer 5′-ggatccTCAGTCGACCTTTTTTACG­GGGCGCAGGC-3′ (the lower-case sequences correspond to the *Nde*I/*Bam*HI restriction sites used for cloning). The PCR product was ligated into PCR-BluntII-TOPO using the Zero Blunt TOPO Cloning Kit (Invitrogen) and then cloned into a pET15b (Novagen) cloning vector modified to encode a tobacco etch virus (TEV) protease cleavage site in place of the thrombin protease cleavage site. The construct was verified by DNA sequencing (DNA Sequencing Unit, University of Dundee).

The recombinant protein was produced in *Escherichia coli* BL21 (DE3) pLysS cells (Stratagene). Cultures were grown for 3 h at 310 K in auto-induction medium (Studier, 2005 ▶) supplemented with 50 µg l^−1^ carbenicillin before overnight growth at 295 K. Cells were harvested by centrifugation (3500*g* at 277 K for 30 min). The cell pellet was resuspended in buffer *A* (25 m*M* Tris–HCl pH 7.5, 500 m*M* NaCl, 20 m*M* imidazole pH 8.5) supplemented with an EDTA-free Protease Inhibitor Cocktail Tablet (Roche) and 0.2 mg DNase I (Sigma–Aldrich). Cells were lysed using a continuous-flow cell disrupter (Constant Systems) at 207 MPa and cell debris was removed following centrifugation (40 000*g* at 277 K for 30 min). *Sm*Lip was purified using nickel-affinity chromatography with a 5 ml HisTrap HP column (GE Healthcare) pre-charged with Ni^2+^. A step gradient of 5% buffer *B* (25 m*M* Tris–HCl pH 7.5, 500 m*M* NaCl, 500 m*M* imidazole) was used to remove histidine-rich proteins. A linear concentration gradient of imidazole from 5 to 50% buffer *B* was applied to elute the product, which was then dialyzed against buffer *C* (25 m*M* Tris–HCl, 250 m*M* NaCl pH 7.5) at 277 K overnight in the presence of His-tagged TEV protease. The resulting mixture was applied onto the HisTrap column, which bound the cleaved His tag, TEV protease and uncleaved *Sm*Lip. The *Sm*Lip sample from which the His tag had been cleaved was present in the flowthrough. Fractions were analyzed using SDS–PAGE and those containing *Sm*Lip were pooled. The protein was further purified by size-exclusion chromatography using a Superdex 75 26/60 column (GE Healthcare) equilibrated with buffer *C* on an ÄKTApurifier (GE Healthcare). The column had previously been calibrated with the molecular-weight standards blue dextran (>2000 kDa), thyro­globulin (669 kDa), ferritin (440 kDa), aldolase (158 kDa), conalbumin (75 kDa), ovalbumin (43 kDa), carbonic anhydrase (29.5 kDa), ribonuclease A (13.7 kDa) and aprotinin (6.5 kDa) (GE Healthcare; data not shown). The protein eluted as one peak of approximate mass 17 kDa, corresponding to a monomer. Fractions containing the protein were pooled and concentrated to 10 mg ml^−1^ using Amicon Ultra devices (Millipore) for subsequent use. The purity of the protein was confirmed by SDS–PAGE and mass spectrometry (Fingerprint Proteomics Facility, University of Dundee). A theoretical extinction co­efficient of 16 960 *M* ^−1^ cm^−1^ at 280 nm was used to estimate the protein concentration (*ProtParam*; Gasteiger *et al.*, 2005 ▶); the theoretical mass of one subunit was estimated as 16.1 kDa with a calculated isoelectric point of 5.4. The purified protein sample was stored at 277 K until further use.

### 2.2. Crystallization, data collection and structure determination

Initial crystallization screens were carried out at 293 K by the sitting-drop vapour-diffusion method in 96-well plates. This was achieved using a Phoenix liquid-handling system (Rigaku, Art Robbins Instruments) and the commercially available PEG (Qiagen) and JCSG+ (Molecular Dimensions) screens. Crystallization occurred in two conditions, which were further optimized using the hanging-drop vapour-diffusion method with drops consisting of 1 µl protein solution at 10 mg ml^−1^ in 25 m*M* Tris–HCl pH 7.5, 250 m*M* NaCl and 1 µl reservoir solution. The two conditions involved reservoirs consisting of 20% polyethylene glycol 3350, 200 m*M* KI and of 15% polyethylene glycol 3350, 200 m*M* NaCl. Monoclinic blocks with minimum dimensions of approximately 0.3 mm grew over 2 d and the addition of glycerol to 10% proved to be a suitable cryoprotectant.

Crystals from the iodide-containing condition were characterized first and data set I was measured in-house using a Rigaku MicroMax-007 rotating-anode X-ray generator (Cu *K*α, λ = 1.541 Å) coupled to an R-AXIS IV^++^ image-plate detector. A crystal from the second condition was stored in liquid N_2_ and subsequently used to measure a high-resolution data set (data set II) on beamline ID29 at the European Synchrotron Radiation Facility (ESRF; Grenoble, France) using an ADSC Q315R detector. All data were indexed and integrated using *XDS* (Kabsch, 2010 ▶) and scaled using *SCALA* (Evans, 2006 ▶) from the *CCP*4 program suite (Winn *et al.*, 2011 ▶).

Data set I was used to solve the structure by SAD methods targeting the iodides present in the crystallization conditions and to acquire a fairly complete model. The sites of potential anomalous scattering ions or atoms were identified using *PHENIX* (Adams *et al.*, 2010 ▶) and experimental phases were calculated using *Phaser* (McCoy *et al.*, 2007 ▶). Density modification was carried out using histogram matching, averaging on the basis of noncrystallographic symmetry (NCS), and model building was carried out using *RESOLVE* (Terwilliger, 2003 ▶). NCS restraints were employed in the initial refinement calculations, which were performed using *REFMAC*5 (Mur­shudov *et al.*, 2011 ▶). Inspection of the model and the fit to electron-density and difference density maps was carried out in *Coot* (Emsley *et al.*, 2010 ▶). The analysis then switched to the high-resolution synchrotron data set II when it became available and this was used to complete the refinement. *MolProbity* (Chen *et al.*, 2010 ▶) was used to investigate model geometry in combination with the validation tools provided in *Coot*. Analyses of surface areas and interactions were made using the *PISA* (Krissinel & Henrick, 2007 ▶) web service and secondary-structure analysis was performed using *DSSP* (Kabsch & Sander, 1983 ▶). Crystallographic statistics are summarized in Table 1 ▶.

### 2.3. Bacterial two-hybrid analyses

For generation of the plasmid pSC072, the gene fragment encoding *Sm*Lip amino acids 27–176 was PCR-amplified using primers 5′-TATAgcatgcGTAAAGAGGAGGCTGCATGTC­TTCCGCCAAAAGC-3′ and 5′-TATAtctagaGAGTCGAC­CTTTTTTACGGGGC-3′ and cloned into the vector pUT18 (Karimova *et al.*, 2001 ▶) using *Sph*I and *Xba*I restriction sites. The restriction sites are shown in lower case. For generation of another plasmid, pSC080, the same gene fragment was PCR-amplified using primers 5′-TATAggatccAATGTCTTC­CGCCAAAAGCG-3′ and 5′-TATAggtaccAATGATGACG­ACC­CCTATCGC-3′ and cloned into vector pT25 (Karimova *et al.*, 1998 ▶) using *Bam*HI and *Kpn*I restriction sites (again shown in lower case). Bacterial two-hybrid analyses were performed following established protocols (Karimova *et al.*, 1998 ▶, 2000 ▶). *E. coli* BTH101 was transformed with pSC072 (or pUT18 control) and pSC080 (or pT25 control) and the colour of the resulting transformants was scored on MacConkey media with 0.2% maltose (with a positive result being red). For quantitative measurement of the interaction, β-galacto­sidase assays were performed as described by Murdoch *et al.* (2011 ▶) on double-transformed BTH101 grown at 303 K in Luria–Bertani broth (LB) and permeabilized with toluene. Replicate assays were performed on independent transformants.

### 2.4. Cellular localization of Lip

Wild-type *S. marcescens* strain Db10 and the *lip* mutant SJC10 (Murdoch *et al.*, 2011 ▶) were grown for 8 h at 303 K in LB. Subcellular fractionation was performed following an established method (Hatzixanthis *et al.*, 2003 ▶). In brief, following isolation of clean supernatant by centrifugation, washed cell pellets were resuspended in 50 m*M* Tris–HCl pH 7.5, 40%(*w*/*v*) sucrose at 10 ml per gram of cells. EDTA was then added to 5 m*M* (final concentration) and lysozyme was added to 0.6 mg ml^−1^ before incubation at 310 K for 30 min. Sphaeroplasts were harvested by centrifugation and taken up in an equivalent volume of 50 m*M* Tris–HCl pH 7.5 before French pressure treatment. Following ultracentrifugation of the resultant crude extract, the isolated membranes were again taken up in an equivalent volume of 50 m*M* Tris–HCl pH 7.5. This protocol ensured that equivalent proportions of each cell fraction were assayed. 4 µl of each fraction was mixed with SDS sample buffer (100 m*M* Tris–HCl pH 6.8, 3.2% SDS, 3.2 m*M* EDTA, 16% glycerol, 0.2 mg ml^−1^ Bromophenol blue, 2.5% β-mercaptoethanol) and separated by 15% SDS–PAGE prior to anti-Lip immunoblotting. Whole-cell samples com­paring wild-type *versus* SJC10 were prepared by resuspending cells from 100 ml culture in 100 µl SDS sample buffer and boiling for 5 min prior to loading 6 µl onto the gel. Following SDS–PAGE, proteins were electroblotted onto polyvinylidine fluoride membrane (Millipore). *Sm*Lip was detected by hybridization of the primary antibody polyclonal rabbit anti-­Lip (1:4000) followed by the secondary antibody HRP-conjugated goat anti-rabbit (Thermo; 1:10000) and the use of an enhanced chemiluminescent detection kit (Millipore).

## 3. Results and discussion

### 3.1. Structure determination

Full-length *Sm*Lip consists of 176 residues. A truncated version of *Sm*Lip consisting of an N-terminal hexahistidine tag plus a TEV protease recognition site followed by residues Ala30–Asp176 was obtained in recombinant form and purified. The N-terminal 29 amino acids, which include the lipid­ation signal peptide and the first four residues of the mature protein, have been omitted. This sample gave monoclinic crystals. The asymmetric unit consists of four polypeptide chains, labelled *A*–*D*, with an estimated solvent content of 45% and a *V*_M_ of 2.27 Å^3^ Da^−1^.

Medium-resolution diffraction data were recorded in-house and the anomalous scattering information was used in a SAD approach to phasing. 13 potential iodide positions were identified and produced a figure of merit of 0.43 to 2.35 Å resolution. Subsequently, 12 of these positions were confirmed by refinement with this data set. The initial model constructed in *RESOLVE* consisted of 293 residues, with a correlation coefficient of 0.55 and *R*_work_ and *R*_free_ values of 46% and 49%, respectively. The first round of model building in *Coot* extended this to 467 residues, with a correlation coefficient of 0.72 and *R*_work_ and *R*_free_ values of 33% and 37%, respectively. At this point the high-resolution synchrotron data (1.92 Å resolution) became available and were used to continue the analysis. The refinement proceeded with the release of NCS restraints and the incorporation of water molecules, an Na^+^ ion, ethylene glycol and a number of side chains with dual rotamer conformations. This data set was derived from crystals grown in the presence of chloride instead of iodide. However, we did not assign any chloride ions to the structure, noting that typical water molecules occupy the previously identified iodide-binding sites. The refinement was terminated when there were no significant changes in *R*_work_ and *R*_free_ and inspection of the difference density map suggested that no further corrections or additions were justified. Several dual rotamers are incorporated into the model. Disorder was evident at several positions, for example the N-terminus, where it was not possible to interpret diffuse and weak electron density. Consequently, several residues are absent from the model. Molecule *A* consists of residues 32–173; molecule *B* of residues 33–142 and 147–176; molecule *C* of residues 34–50, 53–143 and 147–175; and molecule *D* of residues 33–50 and 55–175. The geometry of the model is acceptable (Table 1 ▶).

### 3.2. Self-association and localization *in vivo*

Previous work on SciN, the Lip homologue from entero­aggregative *E. coli*, showed that the protein is localized in the outer membrane, facing the periplasm (Aschtgen *et al.*, 2008 ▶). Examination of the amino-acid sequence of the N-­terminus of *Sm*Lip predicts that this is also an outer-membrane lipoprotein. The *LipoP* 1.0 algorithm (Juncker *et al.*, 2003 ▶) predicts that *Sm*Lip has a lipoprotein signal peptide and that signal peptidase II cleavage occurs between Gly25 and Cys26, with the cysteine subsequently being lipidated. Additionally, the residue at the +2 position following cleavage is Met27 (*i.e.* it is not an aspartate, which directs retention in the inner membrane); therefore, *Sm*Lip should proceed to the outer membrane *via* the Lol system (Bos *et al.*, 2007 ▶).

In order to investigate whether *Sm*Lip undergoes self-interaction, the bacterial two-hybrid system (Karimova *et al.*, 2000 ▶) was utilized in *E. coli*. This assay involves reconstitution of adenylate cyclase activity from two non-interacting cyclase fragments, called T18 and T25, from *Bordetella pertussis*. The presence of cyclic AMP activates the transcription of maltose and lactose catabolic operons by *E. coli*. This can be detected by direct measurement of β-galactosidase activity or by using the observation that bacteria capable of fermenting maltose acidify the medium and thus generate a red colour on MacConkey–maltose indicator plates.

**Figure 1:**
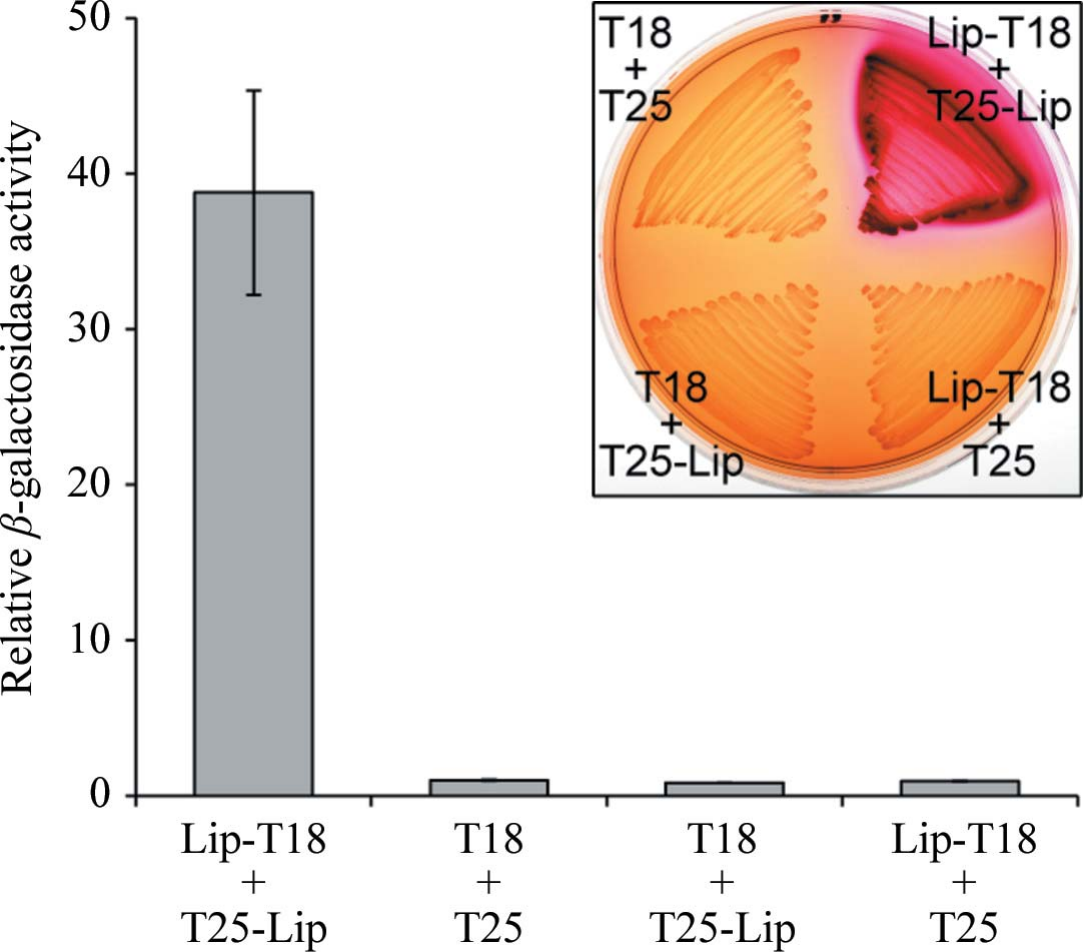
Detection of Lip–Lip self-interaction. The bacterial two-hybrid system was used to detect an *in vivo* interaction between Lip (minus signal peptide) fused to T25 (pSC080) and Lip (minus signal peptide) fused to T18 (pSC072). The empty vectors pUT18 and pT25 represent negative controls. The graph shows the output from the two-hybrid system detected as β-galactosidase activity expressed relative to the pUT18/pT25 baseline level (the maximal β-galactosidase activity observed for the Lip–Lip interaction corresponded to >5000 Miller units). Bars show mean ± SEM. Inset: colourimetric readout of the two-hybrid assay following growth of *E. coli* BTH101 carrying the above plasmids on MacConkey–maltose agar (red is a positive result).

*Sm*Lip was introduced as both bait and prey by encoding on plasmids pUT18 and pT25, and a strong positive result was observed (Fig. 1 ▶). Mature *Sm*Lip (lacking the N-terminal signal peptide) was used for this experiment, firstly to correspond to the form of *Sm*Lip for which the structure was solved and secondly to ensure that both partners were local­ized together in the cytoplasm after fusion with T18 or T25. This positive result indicates that Lip does indeed self-associate within the cell and that neither localization in the outer membrane nor other components of the type VI secretion apparatus are required for self-interaction. We note, however, that this system is unable to distinguish between dimerization or higher order oligomerization.

As a control for any propensity of *Sm*Lip to form non­specific interactions, in addition to the lack of interaction with the T18 and T25 proteins demonstrated in Fig. 1 ▶ we tested whether *Sm*Lip gave a positive bacterial two-hybrid result with several cytoplasmic components of the T6SS (with which, as a periplasmic protein, it should not interact). *Sm*Lip gave a negative result (indistinguishable from the T25 + T18 negative control) when tested against the proteins VipB, TssK and TssL (data not shown).

**Figure 2:**
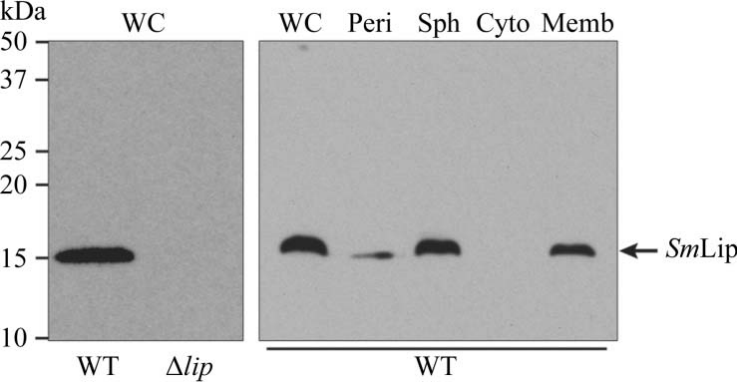
Cellular localization of native *Sm*Lip in *S. marcescens*. Anti-Lip immunoblot of whole cells or cellular fractions prepared from wild-type *S. marcescens* strain Db10 or the Δ*lip* mutant SJC10 (WC, whole cell; Peri, periplasm; Sph, sphaeroplast; Cyto, cytoplasm; Memb, membranes). The predicted size of mature *Sm*Lip is 16 kDa.

In order to confirm the localization of the native Lip protein in *S. marcescens*, we utilized an anti-Lip polyclonal antibody to probe each of the major cellular fractions in this organism. As shown in Fig. 2 ▶, native *Sm*Lip is found exclusively in the membrane fraction, confirming the predicted localization of the protein and the functionality of the signal peptide.

### 3.3. Overall structure

**Figure 3:**
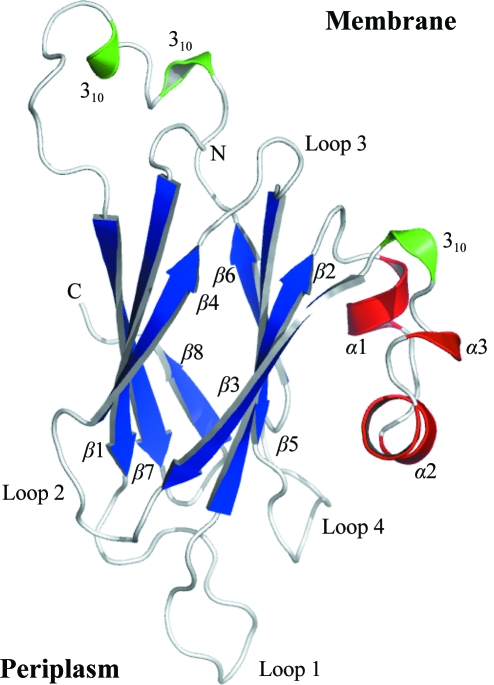
The secondary structure and fold of *Sm*Lip. β-Strands are shown as blue arrows and α-helices and 3_10_-turns as red and green ribbons, respectively. The N- and C-terminal residues are labelled and the orientation of the protein with respect to the outer membrane and periplasm is suggested.

The *Sm*Lip polypeptide can be classified as a new member of the transthyretin-like superfamily and a detailed comparison will be given below. The protein displays a compact globular structure dominated by an eight-stranded β-sandwich (Fig. 3 ▶; Supplementary Fig. S1^1^). The order of the strands is 8–7–1–4 and 6–5–2–3. There are three short α-helical segments and three 3_10_-helix turns. The four *Sm*Lip polypeptide chains in the asymmetric unit are similar, with the root-mean-square (r.m.s.) deviations between superimposed C^α^ atoms ranging from 1.3 Å (monomers *A* and *B*) to 0.8 Å (monomers *A* and *D*) with an average value of 0.95 Å.

**Figure 4:**
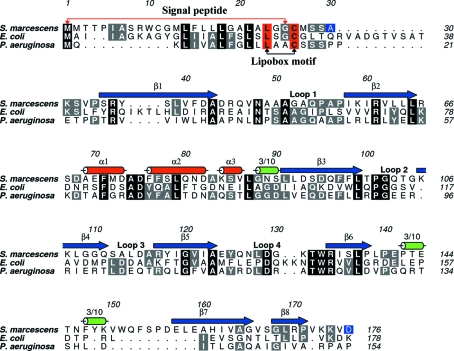
The primary and secondary structure of *Sm*Lip and sequence alignment with two homologues. *S. marcescens* Lip is aligned with the homologous proteins from enteroaggregative *E. coli* (GenBank CBG37366.1) and *P. aeruginosa* (NCBI Reference Sequence NP_248770.1, PA0080). The secondary structure of *Sm*Lip is depicted with blue arrows for β-strands and red and green cylinders for α-­helices and 3_10_-helices, respectively. Residues conserved in all three sequences are shown in black boxes and those conserved in only two sequences are shown  in grey boxes. The start and finish of the lipobox motif are marked by red boxes; the residues at the start and end of the sequence used in the structure analysis (Ala30–Asp176) are shown in blue boxes. The alignment was generated using *T-­Coffee* (Di Tommaso *et al.*, 2011 ▶) in the M-Coffee mode and the figure was prepared using *ALINE* (Bond & Schüttelkopf, 2009 ▶).

Although a set of core conserved proteins are encoded by the T6SS gene clusters in different Gram-negative bacteria (data not shown), there is a large degree of variation in the amino-acid sequences of these proteins. Lip and its ortho­logues, for example, are relatively poorly conserved. Excluding the signal peptide and lipobox motif (Fig. 4 ▶), *Sm*Lip shares only about 20% sequence identity with SciN, the homologue from enteroaggregative *E. coli*. This increases to near 40% in comparison with the homologue from the *P. aeruginosa* HSI-1 T6SS. Sequence conservation is noted in loop 1, near α1 and α2, in loop 2 and in the loop 4–β6 region (Fig. 4 ▶).

An alignment of *Sm*Lip with eight orthologues (Supplementary Fig. S2^1^) reinforces the observation of a low level of sequence identity for this protein. Excluding two residues in the lipobox motif, only six residues are strictly conserved: Asn48, Leu99-*X*-Pro101-Gly102, Gly120 and Ala124. All six residues appear to contribute to the conformation of specific parts of the fold (data not shown). The side chain of Asn48 accepts a hydrogen bond from the main-chain amide of Gln126, helping to define the conformation of loop 4. The Leu99-*X*-Pro101-Gly102 segment defines the structure of the turn after β3 leading into loop 2. Gly120 and Ala124 occur in β5 and contribute hydrogen bonds to form interactions with β2 and β6 on either side. An increase in size of the side chain at either of these positions would be likely to be disruptive to the formation of this β-sheet, which forms one side of the structure. There is no obvious hydrophobic, basic or acidic surface feature on *Sm*Lip that is likely to be conserved within the Lip proteins since the few conserved residues are mainly buried.

The information provided in §[Sec sec3.2]3.2 identifies that the N-­terminus of the structure is placed close to the outer membrane, hence the assignment of the orientation of *Sm*Lip with respect to the outer membrane (Fig. 3 ▶). By extension, we note that the areas of *Sm*Lip in which sequence conservation is observed mainly appear to contribute to stabilizing parts of the structure that jut out into the periplasm. They may therefore serve to define the structure of parts of Lip that are responsible for interaction with other molecules in the periplasm.

### 3.4. The tetramer is likely to be a crystallographic artefact

**Figure 5:**
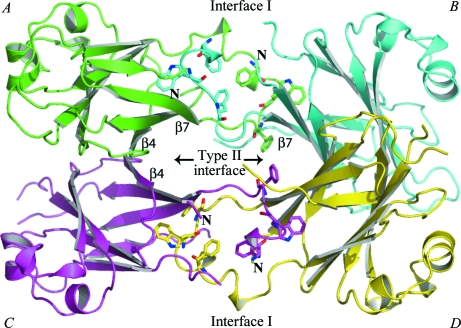
The asymmetric unit. The four molecules that constitute the asymmetric unit are shown in different colours using the secondary-structure assignment given in Fig. 1 ▶ and labelled. The two types of protein–protein interface are labelled, as are the β4 and β7 strands. Residues that contribute significantly to the type I interface (Phe147, Trp151 and Phe153) are depicted as sticks.

Gel-filtration data acquired during purification indicated that *Sm*Lip is a monomer in solution (data not shown). In contrast, the bacterial two-hybrid data revealed a propensity for self-interaction and the asymmetric unit is a tetramer displaying 222 point-group symmetry (Fig. 5 ▶). The accessible surface area (ASA) of the *Sm*Lip polypeptide averages out at approximately 8350 Å^2^; the range is from 8200 Å^2^ for molecule *D* to 8510 Å^2^ for molecule *A*. Each molecule in the asymmetric unit interacts with two of the other three and two types of protein–protein interface are formed between molecules *A*–*B* and *C*–*D* (interface I) and between molecules *A*–*C* and *B*–*D* (interface II). The type I interface, which is larger, covers an area that is approximately 13% of the ASA of the *Sm*Lip molecule. Such coverage certainly indicates potential for a biologically relevant dimer. This interface is primarily formed by the antiparallel alignment of two β7 strands. Three aromatic residues, Phe147, Trp151 and Phe153, contribute van der Waals interactions to the association and, by virtue of their relative bulk, also to the ASA (data not shown). The type II interface covers about 6.5% of the ASA of a molecule, a level typical of the values observed simply owing to molecular packing in a crystal lattice. This interface is formed by the antiparallel alignment of two β4 strands. The areas of *Sm*Lip involved in forming a tetramer are not con­served in the homologues from *E. coli* or *P. aeruginosa* (Fig. 4 ▶) and it is unlikely that such a tetramer is a generic feature of this lipoprotein.

The spatial placement of the N-­terminal residues in the asymmetric unit is such that it is unlikely that an oligomeric assembly could form when the protein is anchored in the membrane by the lipidated Cys26 at the N-terminus. The N-­termini of molecules *A* and *D* are on the same side of the tetrameric assembly but are opposite to those of molecules *B* and *C*. As explained, there are no direct interactions formed between molecules *A* and *D* or molecules *B* and *C*. That the bacterial two-hybrid experiments reveal a propensity for self-interaction of the truncated protein in the cytoplasm is in one sense consistent with the crystal structure of the truncated version of *Sm*Lip, which shows a tetrameric assembly con­taining a plausible dimer. On the other hand, the structure of the tetramer is incompatible with dimeric or tetrameric structures if the N-terminus is membrane-bound. These observations may be a result of the different concentrations and experimental conditions used. We suggest that *Sm*Lip is a membrane-bound monomer but displays a propensity to interact with itself.

A reviewer commented on the possibility that the *Sm*Lip tetramer might represent an inactive or alternative state of the protein. This is an intriguing suggestion and raises questions about how conversion to an active form might occur and how the T6SS itself is regulated. We have no data to address this issue and further studies would be required to investigate such a possibility.

### 3.5. Comparisons with structural homologues

A search for structural neighbours in the Protein Data Bank using the *PDBeFold* (http://pdbe.org/fold) and *ProFunc* servers (Laskowski *et al.*, 2005 ▶) gives a *Z* score of 6.1 with sea bream transthyretin (Eneqvist *et al.*, 2004 ▶; PDB entry 1sn0). This matched 84 residues with an r.m.s.d. of 2.7 Å. The β-sheet structures align well (Supplementary Fig. S3^1^). The r.m.s.d. and relatively low *Z* score reflect the low sequence identity shared between the two proteins of approximately 7%. Nevertheless, the structural relationship is clear and *Sm*Lip can be classed as a new member of the transthyretin-like protein family. The only other member of this protein family is 5-hydroxyisourate hydrolase (EC 3.5.2.17; Hennebry *et al.*, 2006 ▶), an enzyme that is found only in prokaryotes, leading to the conclusion that this represents an example of divergent evolution (Hennebry, 2009 ▶). The sequence identity shared between this hydrolase and *Sm*Lip is only 6%, but the similarity in fold is evident (data not shown). We carried out further comparisons seeking to inform on Lip function.

Transthyretin binds the hormone thyroxine, self-interacts to form a tetramer and also forms a complex with retinol-binding protein (Blake *et al.*, 1978 ▶; Wojtczak *et al.*, 1992 ▶; Monaco *et al.*, 1995 ▶; Zanotti *et al.*, 2008 ▶). In common with transthyretin, *Sm*Lip forms a tetrameric assembly. However, the *Sm*Lip oligomer is distinct and an overlay of one *Sm*Lip polypeptide with a subunit from transthyretin does not produce an overlap of any of the other polypeptides (data not shown).

Transthyretin forms a dimer by antiparallel self-association of the β6 and β8 strands, creating a curved eight-stranded β-­sheet (Blake *et al.*, 1978 ▶). The binding of the hormone thyroxine occurs at the tetramer interface created by the convex surfaces of two of these eight-stranded β-sheets as the protein assembles as a dimer of dimers. The thyroxine-binding residues in transthyretin are not conserved in *Sm*Lip and an overlay of an *Sm*Lip polypeptide and transthyretin subunit places the ligand-binding site on the surface of the former (Supplementary Fig. S3^1^). It is unlikely that *Sm*Lip acts to bind hydrophobic ligands of the type that transthyretin can bind.

Transthyretin associates with retinol-binding protein using residues in three turns: two from one subunit that link β1 to β2 and β4 to β5, and one from another subunit that links β1 to β2 (Monaco *et al.*, 1995 ▶). These parts of the transthyretin structure correspond to loops 1 and 3 of *Sm*Lip. Loop 1 is directed out from the globular fold into the periplasmic space; it is placed to interact with physiological partners and may represent a binding site for other proteins/molecules.

In a recent study of *Klebsiella pneumoniae* 5-hydroxy­isourate hydrolase, the residues important for catalytic function were confirmed as His7, Arg41, His92 and Ser108, which together with Tyr105 form a polar and symmetric active site at a dimer interface (French & Ealick, 2011 ▶). A structure-based sequence alignment matches four of these catalytic residues (with the exception being Ser108) to Asp42, Gly105, His161 and Val172, respectively, in *Sm*Lip. The polypeptides do not overlay in the vicinity of Ser108 (data not shown) and it is unlikely that Lip has any hydrolase activity.

The biological role of *Sm*Lip or its orthologues in the T6SS has yet to be unambiguously defined. Structural comparisons appear to rule out, rather than assign, a function. The pro­pensity to self-associate using parts of the *Sm*Lip structure that will be exposed in the periplasm suggests that this protein, exploiting the lipid anchor, helps to bind and position different components of the secretion apparatus at the outer mem­brane. Future experiments, aided by the structural model, can address this hypothesis.

## Supplementary Material

PDB reference: Lip, 4a1r

Supplementary material file. DOI: 10.1107/S0907444911046300/mn5005sup1.pdf

## Figures and Tables

**Table 1:** Crystallographic statistics Values in parentheses are for the highest resolution shell.

	Data set I	Data set II
Space group	*C*2	*C*2
Wavelength (Å)	1.5418	1.007
Unit-cell parameters (Å, °)	*a* = 139.7, *b* = 77.6,*c* = 54.3, β = 98.4	*a* = 139.7, *b* = 77.8, *c* = 54.5, β = 98.3
Resolution (Å)	19.7–2.35 (2.48–2.35)	39.8–1.92 (2.02–1.92)
No. of reflections recorded	94650 (11192)	280802 (38597)
Unique reflections	23495 (3109)	43478 (6188)
Completeness (%)	98.3 (90.0)	98.2 (95.8)
Multiplicity	4.0 (3.6)	6.5 (6.2)
〈*I*/σ(*I*)〉	30.9 (6.6)	20.6 (3.7)
Anomalous completeness (%)	95.7 (84.5)	—
Anomalous multiplicity	2.0 (1.8)	—
Wilson *B* (Å^2^)	47.7	32.7
No. of residues/waters	—	541/336
*R*_merge_† (%)	2.6 (17.1)	4.9 (44.2)
*R*_work_‡ (%)	—	22.0
*R*_free_§ (%)	—	29.2
Average *B* factors (Å^2^)		
Chain *A*	—	36.1
Chain *B*	—	41.6
Chain *C*	—	49.5
Chain *D*	—	59.8
Waters	—	46.2
Na^+^	—	37.8
Ethylene glycol	—	60.2
Cruickshank DPI¶ (Å)	—	0.2
Ramachandran plot		
Most favoured	—	516 residues
Additional allowed	—	21 residues
Outliers	—	Molecule *D*: Phe97, Asp129; molecule *B*: Pro142, Ser154
R.m.s.d. on ideal values††		
Bond lengths (Å)	—	0.01
Bond angles (°)	—	1.42

† *R*_merge_ = 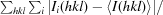 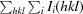, where *I* *_i_*(*hkl*) is the intensity of the *i*th measurement of reflection *hkl* and 〈*I*(*hkl*)〉 is the mean value of *I* *_i_*(*hkl*) for all *i* measurements.

‡ *R*_work_ = 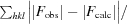 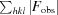, where *F*_obs_ is the observed structure factor and *F*_calc_ is the calculated structure factor.

§ *R*_free_ is the same as *R*_cryst_ except calculated with a subset (5%) of data that were excluded from the refinement calculations.

¶ Cruickshank (1999 ▶).

†† Engh & Huber (1991 ▶).

## Footnotes

1 Supplementary material has been deposited in the IUCr electronic archive (Reference: MN5005). Services for accessing this material are described at the back of the journal.
