# Supplementary material for: The structure of Serratia marcescens Lip, a membrane-bound component of the type VI secretion system
Source: Acta Crystallogr D Biol Crystallogr. 2011 Nov 18;67(Pt 12):1065–72. doi: 10.1107/S0907444911046300 (PMC3225178; doi:10.1107/S0907444911046300)

Supplementary material.

The structure of *Serratia marcescens* Lip; a membrane bound component of the Type VI secretion system.

Vincenzo A. Rao, Sharon M. Shepherd, Grant English, Sarah J. Coulthurst and William N. Hunter*

College of Life Sciences, University of Dundee, DD1 5EH, United Kingdom.

*Correspondence e-mail: w.n.hunter@dundee.ac.uk

Figure S1. The secondary structure and fold of *Sm*Lip. β-strands are shown as arrows, α-helices and 3_{10} turns as ribbons respectively. The N and C terminal residues are labelled and the polypeptide is color ramped from blue at the N-terminus, to red at the C-terminus.

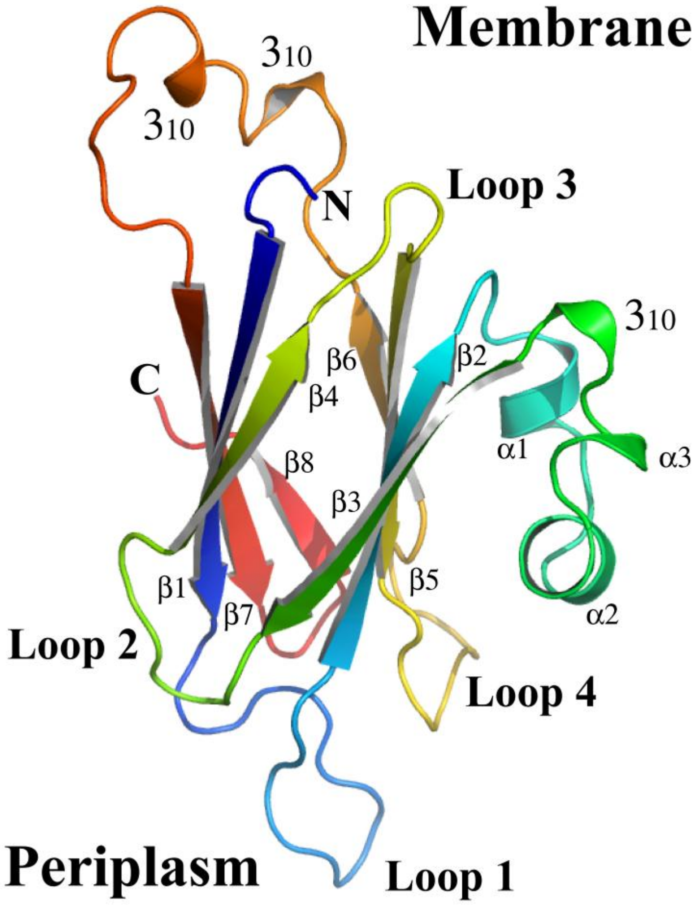

Figure S2. Sequence alignment of *Sm*Lip with eight orthologues. *S. marcescens* Lip is aligned with proteins from *Escherichia coli* (GenBank: CBG37366.1), *Vibrio cholerae* (NCBI Reference Sequence: ZP_01680158.1), *Pseudomonas aeruginosa* (NCBI Reference Sequence: NP_248770.1, PA0080 and NP_250357.1, PA1666), *Burkholderia cenocepacia* (NCBI Reference Sequence: YP_834112.1), *Burkholderia pseudomallei* (NCBI Reference Sequence: YP_112106.1), *Aeromonas hydrophila* (NCBI Reference Sequence: YP_856372.1) and *Edwardsiella tarda* (GenBank: ABW69084.1). Residues conserved in all sequences are enclosed in a cyan block, conserved in all but one sequence in blue, conserved in all but two sequences magenta and conserved in all but three sequences yellow. The start and finish of the lipobox motif are marked, as well as the signal peptide sequence. The alignment was generated the same way as for Figure 2.

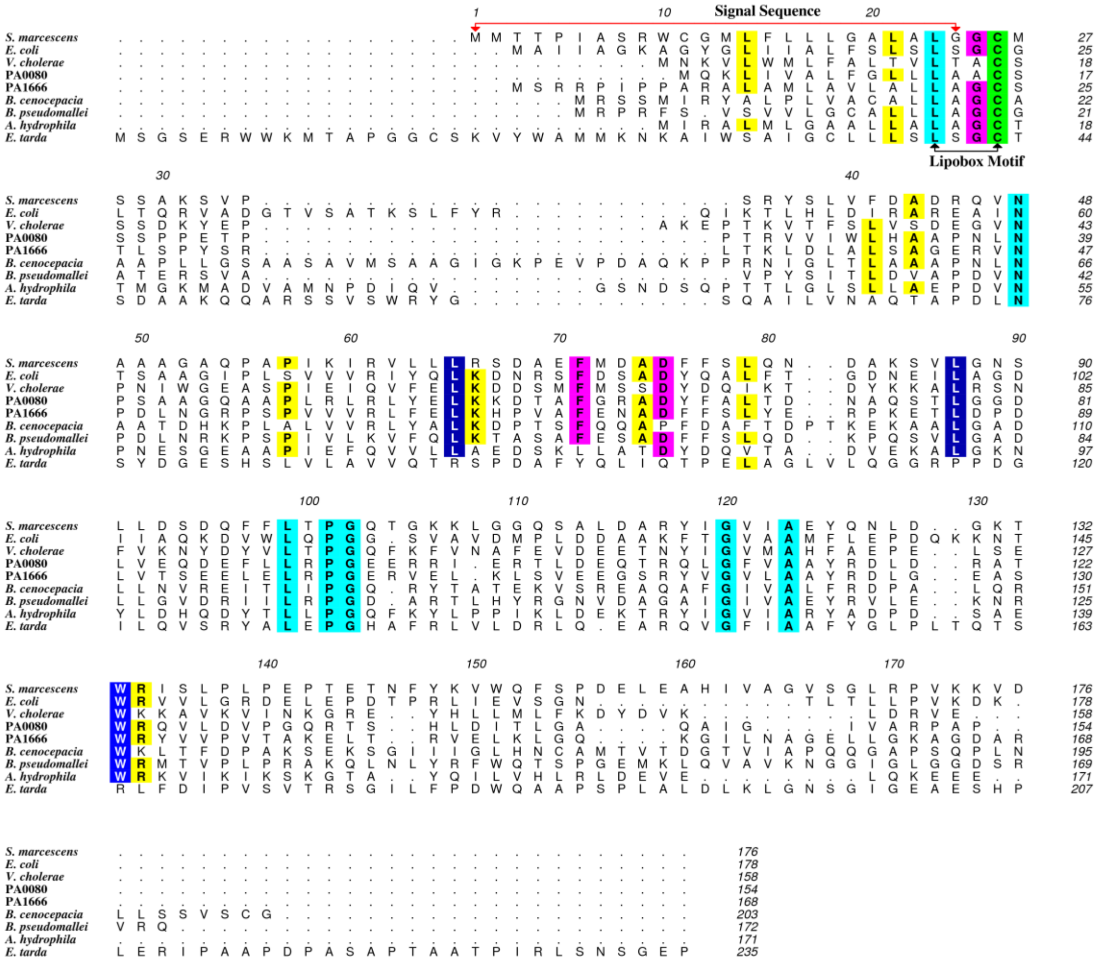

Figure S3. An overlay of *Sm*Lip and a transthyretin subunit complexed with thyroxine. The polypeptide trace for *Sm*Lip is black, transthyretin red. Thyroxine is shown as sticks colored purple for I, grey C, red O and blue N.

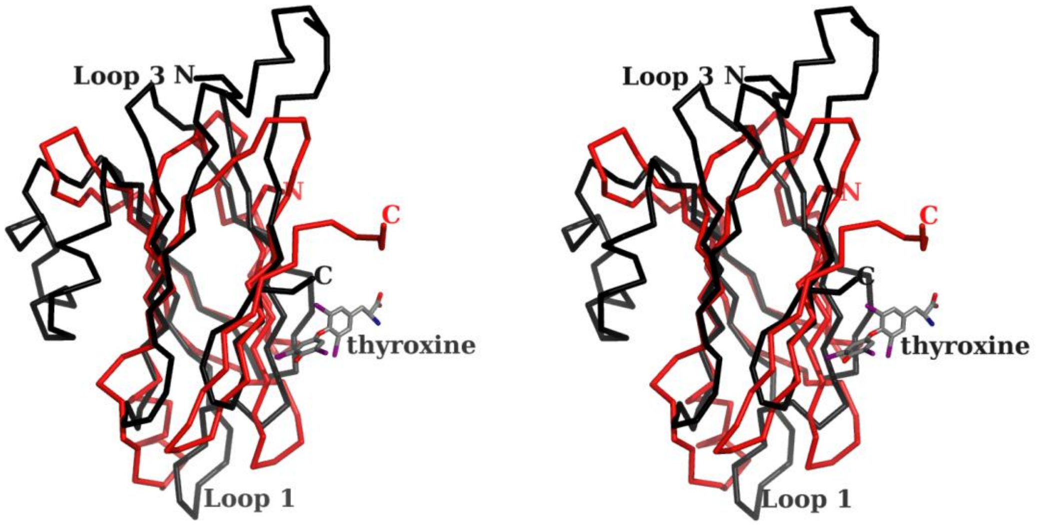

Supplement: Supplementary file 1 [file d-67-01065-sup1.pdf]
